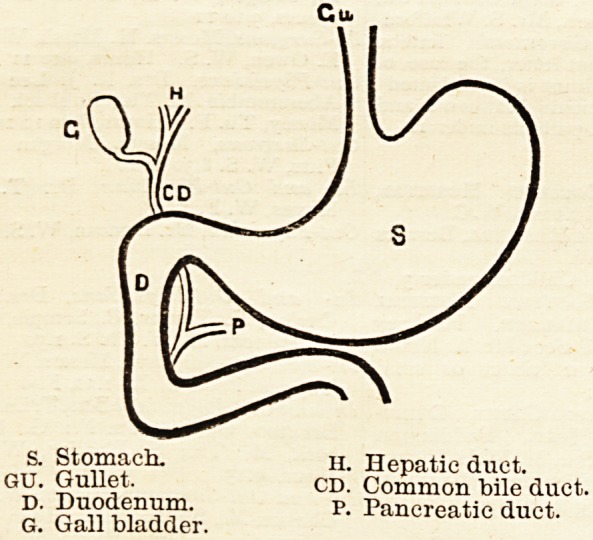# The Stomach

**Published:** 1887-03-12

**Authors:** 


### DISORDERS OF DIGESTION.*

### THE STOMACH.

In a recent paper a short account was given of normal digestion in the mouth. Good teeth, and an abundance of healthy saliva, were there stated to be essential to the first, or mouth part of digestion. From the mouth the food is passed down to the stomach by the gullet. Most people know that the stomach is a bag; but only physiologists know what a wonderful bag it is. Here is a diagrammatic outline of its shape, with a small portion of the duodenum or upper bowel, and the gall bladder and common bile duct; the hepatic and pancreatic ducts are also shown.

S. Stomach.

GU. Gullet.

D. Duodenum.

G. Gall bladder.

H. Hepatic duct.

CD. Common bile duct.

P. Pancreatic duct.

Physiologists are very curious and inquiring persons, and it is probable that some of them would give a good round sum to anybody who would have a window made in the wall of his stomach, so that they might *see* what actually takes place when digestion is going on. We cannot wonder at their eagerness, if we consider for a moment how difficult it is to obtain real and accurate knowledge in any other way. And, indeed, until within a very few years, the knowledge of digestion possessed by even the most scientific physiologists was, more or less, guesswork, and not truly *scientific knowledge* at all. A very lucky accident happened, however, in America some years ago—such a thing never happened, that we know of, either before or since, in the whole world's history. Alexis St. Martin had a hole made into the front wall of his stomach, and a flap of skin could be lifted up at any time in such a way as to expose the inside of the organ. Dr. Beaumont, an American physician, with the promptitude of his nation, took advantage of St. Martin's accident, and employed him, not only as his coachman, but also as his patent digester, so to speak. Whenever the doctor wished, he could open the window in St. Martin's stomach, and observe and note down what happened at any time of the day. He observed the stomach when it was full, and when it was empty; when it had liquids alone in it, and when it had solids; when it had roast-beef, and when it had plum-pudding; and, in fine, under every possible variety of circumstance which the ingenious physiological mind could suggest or desire. Here was a veritable scientific gold-mine. Here was nature at work in her secret places. Here were *facts*—not guesses and hypotheses. Alexis St. Martin is one of the most eminent saints in the doctors' calendar, and all because he happened to have an involuntary window in his stomach. Perhaps there are other and better known saints who have been canonised on less meritorious grounds.

As the result of observations on St. Martin, and of experiments made on animals in a similar way, the processes of digestion in the stomach are now tolerably well known. The walls of this wonderful bag are set thick with innumerable little glands of different kinds, which secrete peculiar and very potent fluids. The bag is also furnished with an apparatus, whereby it can set up most vigorous wriggling movements. When the stomach is empty the glands do not secrete, and no wriggling takes place; but if a piece of meat or bolus of food is put in, it is the same as if hundreds and thousands of little taps were turned on. For if we had sight powerful enough we should see myriads of little rills of gastric juice flowing in from the open mouths of the secreting glands, and at the same time a vigorous and orderly wriggling would be set up. Within a short time a marked change would have taken place in the food. It would all have become more or less liquefied, and some of it would have become completely so, and perfectly ready for immediate absorption and transmission by the blood-stream to the various tissues hungrily waiting for it. Here, again, as in the mouth, it is easily seen that if we are to have a sufficiency of effective gastric juice, we must have plenty of healthy blood to extract it from, vigorous glands to perform the extraction, efficient nerve-centres and nerve-fibres to stimulate and control the process, and adequate nervous and muscular energy to keep up the wriggling action. Without all these there would be no gastric juice, nor could there be any process of mixing, both of which are absolutely indispensable. Digestion in the stomach is, in many respects, similar to the same operation in the mouth; and, in fact, it is but a continuation to a further stage of that very process. In a future paper something will be said about that peculiar enemy to gastronomic enjoyment and content—the Liver.

* *Disorders of Digestion.* By T. LAUDER BRUNTON, M.D., F.R.S. London: Macmillan and Co.

## Figures and Tables

**Figure f1:**